# Cytotoxic Activity of 3,6-Dihydroxyflavone in Human Cervical Cancer Cells and Its Therapeutic Effect on c-Jun N-Terminal Kinase Inhibition

**DOI:** 10.3390/molecules190913200

**Published:** 2014-08-27

**Authors:** Eunjung Lee, Ki-Woong Jeong, Hum Nath Jnawali, Areum Shin, Yong-Seok Heo, Yangmee Kim

**Affiliations:** 1 Department of Bioscience and Biotechnology, Bio-Molecular Informatics Center, Institute of KU Biotechnology, Konkuk University, Seoul 143-701, Korea; E-Mails: eun0808@konkuk.ac.kr (E.L.); znznzn00@konkuk.ac.kr (K.-W.J.); humnath@konkuk.ac.kr (H.N.J.); byeol@konkuk.ac.kr (A.S.); 2 Department of Chemistry of Konkuk University, Seoul 143-701, Korea; E-Mail: ysheo@konkuk.ac.kr

**Keywords:** anticancer effect, 3,6-dihydroxyflavone, western blotting, binding model, kinase

## Abstract

Previously we have shown that 3,6-dihydroxyflavone (3,6-DHF) is a potent agonist of the human peroxisome proliferator-activated receptor (hPPAR) with cytotoxic effects on human cervical cancer cells. To date, the mechanisms by which 3,6-DHF exerts its antitumor effects on cervical cells have not been clearly defined. Here, we demonstrated that 3,6-DHF exhibits a novel antitumor activity against HeLa cells with IC_50_ values of 25 μM and 9.8 μM after 24 h and 48 h, respectively. We also showed that the anticancer effects of 3,6-DHF are mediated via the toll-like receptor (TLR) 4/CD14, p38 mitogen-activated protein kinase (MAPK), Jun-N terminal kinase (JNK), extracellular-signaling regulated kinase (ERK), and cyclooxygenase (COX)-2 pathways in lipopolysaccharide (LPS)-stimulated RAW264.7 cells. We found that 3,6-DHF showed a similar IC_50_ (113 nM) value to that of the JNK inhibitor, SP600125 (IC_50_ = 118 nM) in a JNK1 kinase assay. Binding studies revealed that 3,6-DHF had a strong binding affinity to JNK1 (1.996 × 105 M^−1^) and that the 6-OH and the carbonyl oxygen of the C ring of 3,6-DHF participated in hydrogen bonding interactions with the carbonyl oxygen and the amide proton of Met111, respectively. Therefore, 3,6-DHF may be a candidate inhibitor of JNKs, with potent anticancer effects.

## 1. Introduction

Cancer can be defined as a disease in which cells grow out of control and spread to surrounding normal tissues. Cancer can be classified as malignant tumors, able to spread by invasion and metastasis. There are numerous known cancers in humans and their occurrence can be attributed to genetic or environmental factors, including chemicals, diet, physical inactivity, infection, radiation, and hormones [1,2,3]. For instance, the most common cause of stomach cancer is *Helicobacter pylori* infection [4]. The lethality rates of several cancers are diminished by aspirin, which is a cyclooxygenase (COX)-2-targeting nonsteroidal drug [5]. In the case of epithelial and stromal cancer cells, including fibroblasts and endothelial cells, inflammatory cells are recruited during cancer progression and proliferation [6]. Cancer-associated fibroblasts play a role in cancer promotion through the secretion of pro-inflammatory factors, thereby contributing to angiogenesis [7]. When epithelial cancer cells stimulate cancer-associated macrophages or fibroblasts, various cytokines and chemokines are secreted into the micro-cancer environment [8]. There have been numerous studies to establish the relationships between cancer and inflammation. The recognition of invading pathogens by toll-like receptors (TLRs) triggers an inflammatory immune response and activates cellular signaling [9]. The mitogen-activated protein kinase (MAPK) signaling pathway is closely linked to extracellular signals that control cellular processes, including cell growth, proliferation, and differentiation, and migration of cancer cells. The MAPK family includes extracellular signal-regulated kinase (ERK), p38 MAPK, and Jun-N terminal kinase (JNK). Among the MAPKs, the JNKs are generally activated by cytokines, UV irradiation, destitution of growth factor, and DNA damage [10]. The tyrosine and threonine residues in the active site of JNK are phosphorylated by mitogen-activated protein kinase kinase (MEK)4 and MEK7 catalysis during JNK activation. The phosphorylation of JNK plays an essential role in cancer suppression related to Ras-induced tumorigenesis [11,12]. Therefore, JNK inhibitors are being considered for drug therapy to treat different cancers. 

Recently, protein kinases have been considered a novel target because of their regulation of cellular functions, such as the cell cycle, and cell proliferation, metabolism, survival, apoptosis, and motility. Protein kinases including c-Src, c-Abl, MAPK, and the epidermal growth factor (EGF) receptor are known to be stimulated in several cancer cells and related to tumorigenesis [13]. Many researchers of cancer treatments focus on searching for kinase inhibitors that could inhibit the interaction of the kinase and substrate or block the kinase’s adenosine triphosphate (ATP) binding site [14].

Polyphenols are found in fruits, vegetables, herbs, and cereals, and many polyphenolic extracts are known to have favorable effects on humans and livestock. Among these polyphenols, flavonoids are secondary metabolites that have been widely shown to have multiple beneficial effects, including antioxidant, antiviral, antibacterial, anti-inflammatory, and anticancer activities [15]. Among the flavonoids, quercetin has been shown to inhibit prostate cancer cell colony formation [16]. In another study, flavonoids interacted with the ATP-binding sites of tyrosine kinases and serine kinases and eventually suppressed their activity [17]. The mechanisms of the inflammatory response of flavonoids have been studied *in vitro* and *in vivo*. The flavonoids, including quercetin, apigenin, luteolin, naringenin, and kaempferol have shown anti-inflammatory activities involving the inhibition of nitric oxide (NO) production in lipopolysaccharide (LPS)- or cytokine-stimulated macrophages [18]. In our previous study, we observed remarkable anti-inflammatory activities in amentoflavone, isolated from *Ginkgo biloba* and *Hypericum perforatum*, and systematically identified the signaling mechanism that modulated this process [19].

In our previous study, we identified that 3,6-dihydroxyflavone (3,6-DHF) is an effective agonist of human peroxisome proliferator-activated receptor gamma (hPPARγ), and is capable of modulating the growth, apoptosis, and differentiation of numerous human cancer cells [20]. We aimed to examine the effects of 3,6-DHF on the proliferation of five human cancer cell lines, namely, human cervical cancer HeLa cells, human breast cancer MCF-7, human metastatic breast carcinoma MDA-MB-231 cells, human lung cancer A549 cells, and human prostate cancer PC3 cells. 3,6-DHF exhibited potent effects on both HeLa and PC3 cells, with IC_50_ values of 12.5 μM and 50 μM, respectively. It has been reported that 3,6-DHF also increases intracellular oxidative stress and lipid peroxidation, thereby affecting the physical and functional properties of the plasma membrane and inducing apoptosis by modulating a caspase cascade [21]. However, studies have not been performed on the role and mechanisms of action of 3,6-DHF in mouse macrophages and human cervical cancer cells.

In this study, the anticancer activities of 3,6-DHF and its mode of action were investigated to evaluate its efficacy as an antitumor drug or chemopreventive supplement. Studies were also performed to investigate the interactions between JNK1 and 3,6-DHF using fluorescence-quenching analysis and docking studies in order to elucidate the role of 3,6-DHF in the direct modulation of JNK1.

## 2. Results and Discussion

### 2.1. Cytotoxicity in HaCaT and NIH3T3 Cells and Anticancer Activity in HeLa Cells

**Figure 1:**
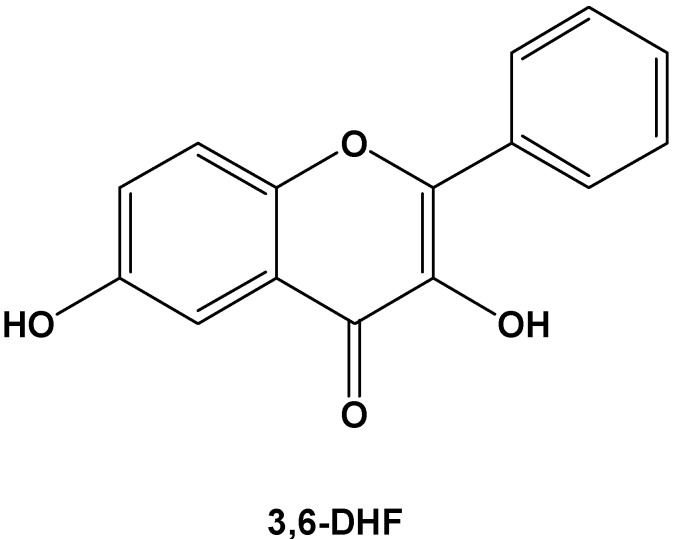
Chemical structure of the flavonoid 3,6-dihydroxyflavone (3,6-DHF).

We examined the anticancer activities of 3,6-DHF (Figure 1) and investigated its mechanism of action *in vitro*.

**Figure 2:**
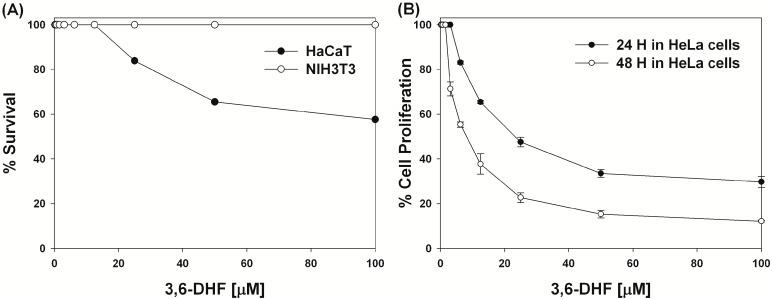
Cytotoxicities of 3,6-dihydroxyflavone (3,6-DHF). (**A**) Concentration-response curves of 3,6-DHF for cytotoxicity toward HaCaT and NIH3T3 cells. (**B**) Concentration-response curves of 3,6-DHF-treated HeLa cells after 24 h and 48 h.

First, in order to determine the nontoxic concentration of 3,6-DHF that could be used for HaCaT and NIH3T3 cells, we investigated its cytotoxicity using a 3-(4,5-dimethylthiazol-2-yl)-2,5-diphenyltetrazolium bromide (MTT) assay (Figure 2A). Concentrations of 3,6-DHF up to 12.5 μM did not affect cell viability. Even in HaCaT cells, the survival rate at concentrations of 50 μM was greater than 50%. Interestingly, concentrations of up to 100 μM did not affect the survival of NIH3T3 cells. We then conducted an MTT assay after 24 h and 48 h incubation with 3,6-DHF and analyzed the proliferation of human cervical cancer HeLa cells. The antitumor effect of 3,6-DHF is shown in Figure 2B. 3,6-DHF had a potent effect against HeLa cells with IC_50_ values of 25 μM and 9.8 μM after 24 h and 48 h, respectively. The growth of cells was inhibited in a dose-dependent manner after exposure to 3,6-DHF for 24 h and 48 h at concentrations ranging from 0 to 100 μM. These results clearly demonstrate that 3,6-DHF possesses novel antitumor activity against HeLa cells, while having very low or no cytotoxicity against RAW264.7 and NIH3T3 cells.

### 2.2. Effects of 3,6-DHF on COX-2 and MAPK Expression in Human Tumor Necrosis Factor-alpha (hTNF-α)-stimulated HeLa Cells

**Figure 3:**
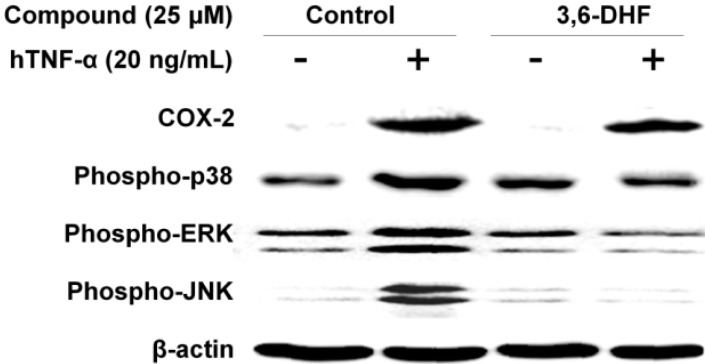
Effects of 3,6-dihydroxyflavone (3,6-DHF) on cyclooxygenase-2 (COX-2), phospho-p38, phospho-extracellular-signal-regulated kinase (ERK), and phospho-Jun-N terminal kinase (JNK) in human tumor necrosis factor alpha (hTNF-α)-stimulated HeLa cells. COX-2, phospho-p38, phospho-ERK, phospho-JNK, and β-actin (loading control) were determined by western blot analysis using specific antibodies. The relative mRNA expression was quantified using Image J (NIH, Bethesda, MD, USA).

Tumor-related inflammatory molecules, including nuclear factor kappa B (NF-κB), interleukin (IL)-1β, IL-6, IL-23, and TNF-α, have been identified in numerous studies [22,23]. Recently, Chang and colleagues demonstrated that 3,6-DHF induces apoptosis by generating reactive oxygen species through activation of the MAPK signaling pathway, as well as by modulating the physical and functional properties of the plasma membrane in human leukemia HL-60 cells [21]. We investigated the mechanisms by which 3,6-DHF inhibited COX-2, and p38/ERK/JNK MAPK expression in HeLa cells stimulated by the pro-inflammatory cytokine hTNF-α. As shown in Figure 3, hTNF-α treatment significantly enhanced the levels of COX-2 and Phospho-p38/ERK/JNK MAPKs in HeLa cells. Treatment of human cervical cancer HeLa cells with 25 μM 3,6-DHF suppressed the phosphorylation of p38, ERK, and JNKs to 42%, 74%, and 97% of the levels in untreated cells, respectively. Our data also revealed that COX-2 protein levels significantly decreased in hTNF-α-stimulated human cervical cancer cells. COX-2 is known to be highly induced at inflammatory and cancer sites in animals and patients with inflammatory and cancer disease [24]. 3,6-DHF treatment suppressed levels of COX-2 to 26%. The results also indicated that hTNF-α induced greater amounts of COX-2, phospho-p38, phospho-ERK, and phospho-JNK in human cervical cancer cells, whereas 3,6-DHF treatment inhibited the levels of these proteins. Western blotting results showed that 3,6-DHF was the major signaling pathway by which hTNF-α induced the synthesis of COX-2, phospho-p38, phospho-ERK, and phospho-JNKs in human cervical cancer cells.

### 2.3. Determination of IC_50_ Values for JNK1 with 3,6-DHF and SP600125 Using the Adenosine Diphosphate (ADP)-Glo Assay

To investigate JNK1 activity in the presence of 3,6-DHF, IC_50_ values were determined based on the results of protein kinase assays performed in the presence of 3,6-DHF and the JNK inhibitor, SP600125. The main goal of these studies was to compare the IC_50_ values of 3,6-DHF and the JNK inhibitor, SP600125. SP600125 is a reversible ATP-competitive inhibitor with more than 20-fold selectivity to JNK1 over other kinases including Erk, p38 MAPKs, MKKs, and PKCs [25]. SP600125, occupied the deepest adenine-binding site only, not expanding to the phosphate-binding site, whereby the binding of SP600125 could not be influenced much by the distortion of the ATP-binding site, which moved the residues of JNK1 interacting with the phosphate groups of ATP far away from the active site [25]. 

**Figure 4:**
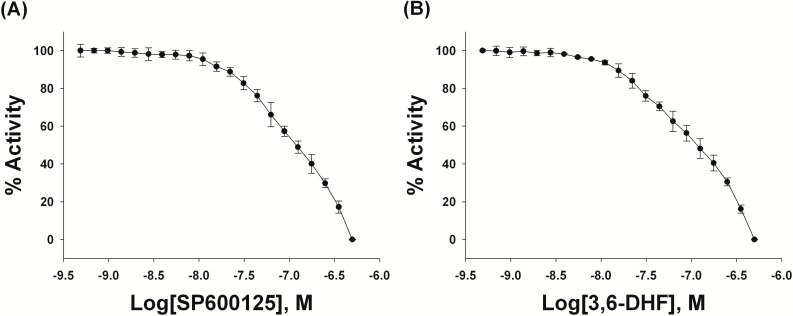
Inhibition of Jun-N terminal kinase 1 (JNK1) by (**A**) SP600125 and by (**B**) 3,6-dihydroxyflavone (3,6-DHF) in an ADP-Glo kinase assay. Each experiment was performed in triplicate.

Initially, the effect of 3,6-DHF on the protein kinase JNK1 was examined. The ADP-Glo assay showed a significant decrease in JNK1 activity with increasing SP600125 and 3,6-DHF concentration as shown in Figure 4A,B, respectively. The respective IC_50_ values of 113 nM and 118 nM were obtained for SP600125 and 3,6-DHF. Therefore, these data show that the ADP-Glo assay-generated IC_50_ values for 3,6-DHF and SP600125 are similar. Therefore, 3,6-DHF, like SP600125, could be a potent JNK1 inhibitor.

### 2.4. Binding Affinities of 3,6-DHF for JNK1

**Figure 5:**
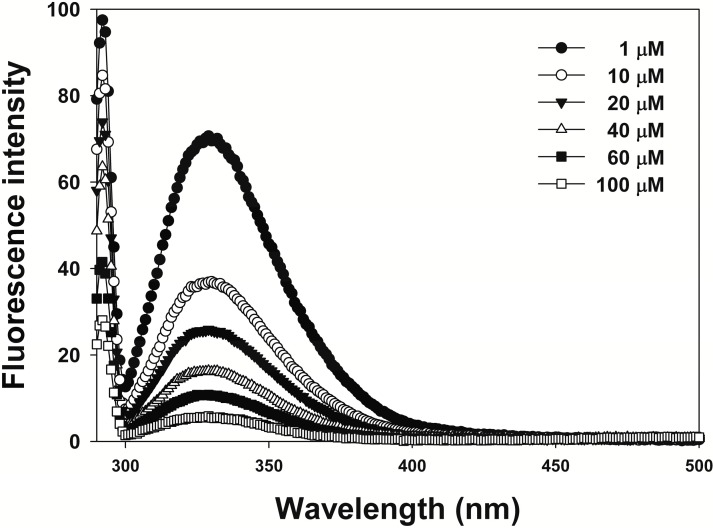
Fluorescence spectra of Jun-N terminal kinase 1 (JNK1) in the presence of 3,6-dihydroxyflavone (3,6-DHF) (0, 10, 20, 40, 60, and 100 μM) at pH 7.0. The sample was excited at 290 nm, and the emission spectra recorded for light scattering effect at 290 to 600 nm.

To investigate the interactions between 3,6-DHF and JNKs, we assessed the binding constants for 3,6-DHF binding to JNK1 using fluorescence-quenching experiments. As shown in Figure 5, the fluorescence intensity was altered in line with increasing 3,6-DHF concentration. 3,6-DHF exhibited good binding affinity to JNK1 at 1.996 × 10^5^ M^−1^, with antitumor effects.

### 2.5. Docking Results of JNK1 and 3,6-DHF

**Figure 6:**
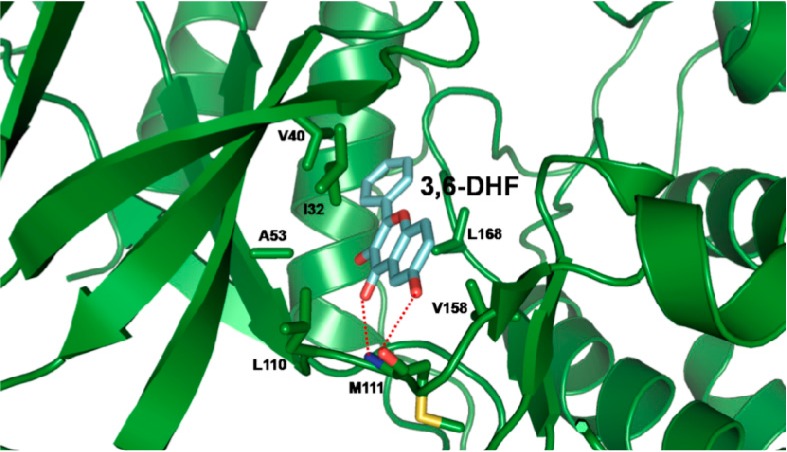
Docking model of 3,6-dihydroxyflavone (3,6-DHF) and Jun-N terminal kinase 1 (JNK1). Hydrogen bonds are depicted as red dashed lines.

To determine the potency of 3,6-DHF as an inhibitor of JNK1; we conducted docking studies with 3,6-DHF and JNK1 in order to develop a binding model for 3,6-DHF and JNK1. The results from the docking studies showed that 3,6-DHF formed two hydrogen bonds with the amide proton and the carbonyl oxygen of Met111 at the ATP active site. As shown in Figure 6; the 6-OH of 3,6-DHF participated in hydrogen-bonding interactions with the carbonyl oxygen of Met111 and the carbonyl oxygen of the C-ring; and formed a hydrogen bond with the amide proton of Met111. In the opposite way; 3,6-DHF might have formed two hydrogen bonds with Lys55 and Asp169. However; this interaction showed a slightly higher CDOCKER energy than the hydrogen bond with Met111. Additionally; 3,6-DHF was involved in further possible hydrophobic interactions with the residues in the binding site; including Ile32; Val40; Ala53; Leu110; Val158; and Leu168. The B-ring of 3,6-DHF participated in a hydrophobic interaction with Val40 and Leu168; and the A-ring of the 3,6-DHF formed hydrophobic interactions with Ile32; Leu110 and Val158. The C-ring of 3,6-DHF was involved in a possible hydrophobic interaction with Ala53. These interactions could contribute to the increase in the binding affinity of 3,6-DHF for JNK1. This binding model is in agreement with the reported models for the binding of SP600125; quercetagetin and rhamnetin with JNK1 [25,26,27]. SP600125 makes two hydrogen bonds with the backbone amide group of Met111; and with the backbone carbonyl oxygen atom of Glu109 in JNK1. The results of this study will be helpful in understanding the mechanism of 3,6-DHF against JNKs.

## 3. Experimental

### 3.1. Reagents

3,6-DHF was obtained from the Indofine Chemical Company (Hillsborough, NJ, USA); it was dissolved in H_2_O:dimethyl sulfoxide (DMSO) (9:1, v/v) at 10 mg/mL in order to prepare the stock solution.

### 3.2. Cytotoxicity against Mammalian Cells

The mouse embryonic fibroblast NIH3T3 and human keratinocyte HaCaT cells were purchased from the American Type Culture Collection (Manassas, VA, USA). The cytotoxicity of 3,6-DHF against NIH3T3 and HaCaT cells was determined using an MTT assay as reported previously [28]. The absorbance at 570 nm was measured using an enzyme-linked immunosorbent assay (ELISA) reader (Molecular Devices, Sunnyvale, CA, USA). Cell survival, expressed as a percentage, was calculated as the ratio of A_570_ for cells treated with 3,6-DHF to those of cells only.

### 3.3. Anticancer Activity against Mammalian Cells

The human cervical cancer HeLa cell line was purchased from the American Type Culture Collection. Cells were cultured in Dulbecco's modifid Eagle’s medium (DMEM) supplemented with 10% fetal bovine serum (FBS) and antibiotic solution (100 units/mL penicillin and 100 μg/mL streptomycin) at 37 °C in a humidified 5% CO_2_ incubator atmosphere. Cells were seeded on 96-well microplates at a density of 1 × 10^4^ cells/well and incubated for 24 h at 37 °C. Serial 2-fold dilutions of 3,6-DHF (100 μL) in DMEM medium were added and incubated for 24 h and 48 h at 37 °C. The anti-tumor activity of 3,6-DHF against HeLa cells was determined using an MTT assay. The absorbance at 570 nm was measured using an ELISA reader (Molecular Devices, Sunnyvale, CA, USA). Cell survival, expressed as a percentage, was calculated as the ratio of A_570_ for cells treated with 3,6-DHF to that of cells only.

### 3.4. Western Blots

Protein was isolated from hTNF-α-stimulated HeLa cells in the presence or absence of 3,6-DHF. Proteins were then detected as reported previously [29,30] using the following antibodies: COX-2 (1:2000; Cell Signaling Technology, Beverly, MA, USA), phospho-ERK (1:2000; Cell Signaling Technology), phospho-p38 (1:2000, Cell Signaling Technology), JNKs and COX-2 (1:1000, Cell Signaling Technology), and β-actin (1:5000, Sigma-Aldrich, St. Louis, MO, USA). The relative amount of protein associated with each antibody was quantified using ImageJ (NIH, Bethesda, MD, USA).

### 3.5. Construction of Expression Plasmids Expressing JNK1

JNKs have numerous isoforms, including JNK1, JNK2, and JNK3, which have been identified in mammals. JNK1 and JNK2 are expressed in the whole cells and tissues of mammals, while JNK3 is found primarily in the brain [31]. To express JNK1, the C-terminal truncated form of human JNK1α1 (residues 1–364) was cloned into the pET21b expression vector from Novagen (Billerica, MA, USA) and expressed in *Escherichia coli* as a 6 His-tagged form at the C-terminus. JNK1 was then purified as reported previously [25].

### 3.6. Measurement of Protein Kinase Activity Using ADP-Glo Kinase Assay

ADP generation was measured with protein kinase assays using the luminescent GDP-Glo assay kit from Promega (Madison, WI, USA). The increase of the luminescence signal indicates the generation of ADP by the protein kinase reaction. We performed the protein kinase assays according to the following assay protocol: Serial 2-fold dilutions of 3,6-DHF and SP600125 as a JNK inhibitor in 1× kinase reaction buffer were prepared and mixed with 20 nM JNK1 and 10 μM ATP. This assay was initiated by incubating the reaction mixture in a 96-well white culture plate at 30 °C for 30 min. After the incubation period, ADP-Glo reagent was added to terminate the kinase reaction. The 96-well white culture plate was incubated for 40 min at room temperature. The kinase detection reagent was then added to convert ADP to ATP and introduce luciferase to detect ATP. The 96-well white reaction plate was incubated for 30 min and then the luminescence was read with a plate-reading luminometer.

### 3.7. Fluorescence Quenching

We titrated 3,6-DHF to 10 μM JNK1 protein solution in 50 mM sodium phosphate buffer containing 100 mM NaCl at pH 8.0, with a final JNK1:3,6-DHF ratio of 1:10. The sample was placed in a 2 mL cuvette, with excitation and emission path lengths of 10 nm. Using tryptophan emission, we determined the fluorescence quantum yields of JNK1 and 3,6-DHF. The methods were performed as described previously [32].

### 3.8. Docking Study

Using the X-ray crystallography structure of JNK1 (3v3v.pdb), we defined the ATP-binding site of JNK1 [31]. 3,6-DHF was docked to JNK1 using CDOCKER, a CHARMm-based molecular dynamics (MD) method for ligand-docking in Discovery Studio modeling (Accelrys Inc., San Diego, CA, USA). This algorithm assumes a rigid protein and permits only the ligand to be flexible. The Input Site Sphere parameter specifies a sphere around the center of the binding site, where the CDOCKER experiment is to be performed. The center of the sphere is used in the CDOCKER algorithm for initial ligand placement. The MD-simulated annealing process is performed using a rigid protein and flexible ligand. The final minimization step is applied to the ligand’s docking pose. The minimization consists of 50 steps of steepest descent followed by up to 200 steps of the conjugated gradient by using an energy tolerance of 0.001 kcal/mol [27,33].

## 4. Conclusions

We found that 3,6-DHF could regulate hTNF-α induced MAPK production by inhibiting the p38/ERK/JNK MAPK-, and COX-2-dependent pathways in human cervical cells. Other studies have shown that treatment of COX-2-over-expressing HT29 human colon cancer cells with 30–120 μM fisetin causes the induction of apoptosis and down-regulation of COX-2 expression without affecting COX-1, and suppresses the secretion of prostaglandin E2 [34]. The flavonoid butein has been reported to show anti-inflammatory and anti-cancer effects. Following butein treatment of A549 lung cancer cells, quantitative PCR and western blot data demonstrated that COX-2 expression was suppressed at the mRNA and protein levels, respectively, and cell-cycle arrest and apoptosis were induced. In this study, butein was suggested as a candidate drug for lung cancer treatment [35]. Quercetin, which is a natural flavonoid, also showed COX-2 inhibitory effects, similar to those of commercially available COX-2 inhibitors (NS-398 and nimesulide), on cell proliferation, apoptosis, PGE2 production, and COX-2 mRNA expression in the OE33 human oesophageal adenocarcinoma cell line [36]. We also found that 3,6-DHF was a potent inhibitor of JNK-dependent cancer in mammalian cells, without causing cytotoxicity. Our binding studies revealed that hydrogen bonding interactions as well as extensive hydrophobic interactions between 3,6-DHF and JNK1 might be essential for the potency of 3,6-DHF as an inhibitor of JNK1, resulting in its anticancer activities.
